# *Vital Signs:* Influenza Hospitalizations and Vaccination Coverage by Race and Ethnicity—United States, 2009–10 Through 2021–22 Influenza Seasons

**DOI:** 10.15585/mmwr.mm7143e1

**Published:** 2022-10-28

**Authors:** Carla L. Black, Alissa O’Halloran, Mei-Chuan Hung, Anup Srivastav, Peng-jun Lu, Shikha Garg, Michael Jhung, Alicia Fry, Tara C. Jatlaoui, Elizabeth Davenport, Erin Burns, Influenza-Associated Hospitalization Surveillance Network, Influenza-Associated Hospitalization Surveillance Network, Arthur Reingold, Nisha B. Alden, Kimberly Yousey-Hindes, Evan J. Anderson, Patricia A. Ryan, Sue Kim, Melissa McMahon, Molly Bleecker, Nancy Spina, Nancy M. Bennett, Krista Lung, Melissa Sutton, William Schaffner, H. Keipp Talbot, Melanie T. Crossland, Carrie Reed, Rachel Holstein, Dawud Ujamaa, Charisse Cummings

**Affiliations:** ^1^Immunization Services Division, National Center for Immunization and Respiratory Diseases, CDC; ^2^Influenza Division, National Center for Immunization and Respiratory Diseases, CDC; ^3^Leidos, Inc, Atlanta, Georgia.; University of California Berkley, California Emerging Infections Program, Oakland, California; Colorado Department of Public Health and Environment; Connecticut Emerging Infections Program, Yale School of Public Health, New Haven, Connecticut; Departments of Pediatrics and Medicine, Emory University School of Medicine, Georgia Emerging Infections Program, Atlanta Veterans Affairs Medical Center, Atlanta, Georgia;; Maryland Department of Health; Michigan Department of Health and Human Services; Minnesota Department of Health; New Mexico Department of Health; New York State Health Department; University of Rochester School of Medicine and Dentistry, Rochester, New York; Ohio Department of Health; Public Health Division, Oregon Health Authority; Department of Health Policy, Vanderbilt University Medical Center, Nashville, Tennessee; Department of Health Policy, Vanderbilt University Medical Center, Nashville, Tennessee; Salt Lake County Health Department, Salt Lake City, Utah; Influenza Division, CDC; Influenza Division, CDC; Influenza Division, CDC; Influenza Division, CDC.

## Abstract

**Introduction:**

CDC estimates that influenza resulted in 9–41 million illnesses, 140,000–710,000 hospitalizations, and 12,000–52,000 deaths annually during 2010–2020. Persons from some racial and ethnic minority groups have historically experienced higher rates of severe influenza and had lower influenza vaccination coverage compared with non-Hispanic White (White) persons. This report examines influenza hospitalization and vaccination rates by race and ethnicity during a 12–13-year period (through the 2021–22 influenza season).

**Methods:**

Data from population-based surveillance for laboratory-confirmed influenza-associated hospitalizations in selected states participating in the Influenza-Associated Hospitalization Surveillance Network (FluSurv-NET) from the 2009–10 through 2021–22 influenza seasons (excluding 2020–21) and influenza vaccination coverage data from the Behavioral Risk Factor Surveillance System (BRFSS) from the 2010–11 through 2021–22 influenza seasons were analyzed by race and ethnicity.

**Results:**

From 2009–10 through 2021–22, age-adjusted influenza hospitalization rates (hospitalizations per 100,000 population) were higher among non-Hispanic Black (Black) (rate ratio [RR] = 1.8), American Indian or Alaska Native (AI/AN; RR = 1.3), and Hispanic (RR = 1.2) adults, compared with the rate among White adults. During the 2021–22 season, influenza vaccination coverage was lower among Hispanic (37.9%), AI/AN (40.9%), Black (42.0%), and other/multiple race (42.6%) adults compared with that among White (53.9%) and non-Hispanic Asian (Asian) (54.2%) adults; coverage has been consistently higher among White and Asian adults compared with that among Black and Hispanic adults since the 2010–11 season. The disparity in vaccination coverage by race and ethnicity was present among those who reported having medical insurance, a personal health care provider, and a routine medical checkup in the past year.

**Conclusions and Implications for Public Health Practice:**

Racial and ethnic disparities in influenza disease severity and influenza vaccination coverage persist. Health care providers should assess patient vaccination status at all medical visits and offer (or provide a referral for) all recommended vaccines. Tailored programmatic efforts to provide influenza vaccination through nontraditional settings, along with national and community-level efforts to improve awareness of the importance of influenza vaccination in preventing illness, hospitalization, and death among racial and ethnic minority communities might help address health care access barriers and improve vaccine confidence, leading to decreases in disparities in influenza vaccination coverage and disease severity.

## Introduction

Influenza is a contagious respiratory disease that can lead to serious illness, hospitalization, and death. CDC estimates that influenza resulted in 9–41 million illnesses, 140,000–710,000 hospitalizations, and 12,000–52,000 deaths annually during 2010–2020 (*1*,*2*). Annual vaccination against seasonal influenza is recommended for all persons aged ≥6 months except when contraindicated (*3*). Vaccination provides important protection from influenza illness and its potential complications. For example, during the 2019–20 season, influenza vaccination prevented an estimated 7.5 million influenza illnesses, 105,000 influenza-associated hospitalizations, and 6,300 influenza-associated deaths (*4*). Persons from some racial and ethnic minority groups experience higher rates of severe influenza and have lower influenza vaccination coverage rates compared with White persons (*5*,*6*). This report presents 1) influenza hospitalization rates by race and ethnicity from the 2009–10 through 2021–22 seasons; 2) trends in influenza vaccination coverage by race and ethnicity from the 2010–11 through 2021–22 seasons; and 3) influenza vaccination coverage stratified by race and ethnicity and health care access variables for the 2021–22 season and possible reasons for observed disparities.

## Methods

**FluSurv-NET.** The Influenza-Associated Hospitalization Surveillance Network (FluSurv-NET) has been previously described (*5*,*7*). Briefly, FluSurv-NET conducts all-age, population-based surveillance for laboratory-confirmed influenza-associated hospitalizations in selected states representing approximately 8%–9% of the U.S. population. Persons met the FluSurv-NET case definition if they resided in the FluSurv-NET catchment area,* were admitted to a hospital during October 1–April 30^†^ and received a positive influenza test result ≤14 days before hospitalization or during hospitalization. Cases in persons aged <18 years and cases from the 2020–21 season were excluded from this analysis because case counts during this season were too low to calculate rates by race and ethnicity.

Population denominators used for rate estimation were obtained from the National Center for Health Statistics.^§^ Unadjusted rates, stratified by age group (18–49, 50–64, 65–74, and ≥75 years), race and ethnicity,^¶^ and influenza season were calculated by dividing the number of hospitalizations by the total catchment population. Unadjusted rates by race, ethnicity, and age group were multiplied by the age distribution of the total FluSurv-NET catchment population to obtain age-adjusted rates; the age groups referenced previously were used for the age adjustment. For rates and rate ratios (RRs), 95% CIs were calculated assuming a simple random sample design and a normal distribution via the SAS STDRATE procedure. All analyses were conducted using SAS software (version 9.4; SAS Institute).

**Influenza Vaccination Coverage.** The Behavioral Risk Factor Surveillance System (BRFSS) is a state-based random-digit–dialed cellular and landline telephone survey that collects information on various health conditions and risk behaviors from one randomly selected adult aged ≥18 years in a household.** BRFSS data for adults aged ≥18 years were analyzed to estimate influenza vaccination coverage for the 2010–11 through 2021–22 influenza seasons. The analysis includes data collected from interviews completed during September–June of each season and vaccine doses received during July–May. Respondents were asked if they had received an influenza vaccine in the past 12 months, and if so, in which month and year. Vaccination coverage estimates were calculated using Kaplan-Meier survival analysis, as previously described (*6*). For the 2021–22 season, for which more detailed data stratified by race and ethnicity and access to care variables are presented, vaccination coverage estimates are based on 291,839 completed interviews; 28,007 respondents were excluded from the analysis because there was no information on whether they had received an influenza vaccine in the past 12 months. The median state BRFSS response rate for a complete or partially complete interview was 42.5% for September–December 2021 and 45.4% for January–June 2022. All estimates were weighted and analyzed using SAS (version 9.4; SAS Institute) and SAS-callable SUDAAN (version 11.0.3; RTI International) statistical software to account for the complex survey design. Differences between estimates were determined using t-tests with p-values <0.05 considered statistically significant.

For each data system, activities were reviewed by CDC and were conducted consistent with applicable federal law and CDC policy.^††^ Sites participating in FluSurv-NET obtained approval from their respective state and local institutional review boards, as applicable. The requirement for informed consent was waived per 45 CFR 46.

## Results

**FIGURE 1:**
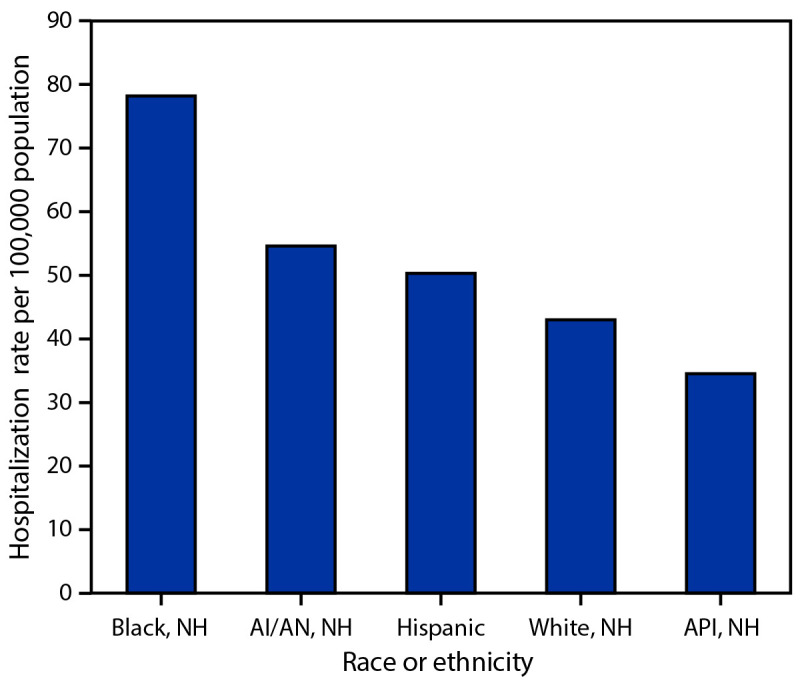
Age-adjusted Influenza-associated hospitalization rates* among adults aged ≥18 years, by race and ethnicity — Influenza-Associated Hospitalization Surveillance Network, United States, 2009–10 through 2021–22^†^ **Abbreviations:** AI/AN = American Indian or Alaska Native; API = Asian or Pacific Islander; NH = non-Hispanic. * Hospitalizations per 100,000 population. ^†^ Excluding 2020–21 season.

**FIGURE 2:**
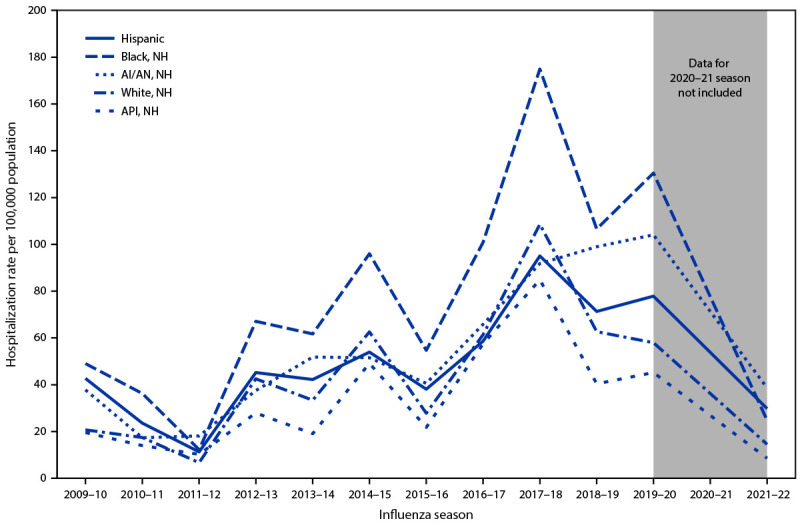
Age-adjusted Influenza-associated hospitalization rates among adults, by race and ethnicity and influenza season — Influenza-Associated Hospitalization Surveillance Network, United States, 2009–10 through 2019–20 and 2021–22* **Abbreviations:** AI/AN = American Indian or Alaska Native; API = Asian or Pacific Islander; NH = non-Hispanic. * Data for 2020–21 season are not included.

From 2009–10 through 2021–22 (excluding 2020–21), age-adjusted influenza-associated hospitalization rates per 100,000 population among adults, by race and ethnicity were as follows: Black, 78.2; AI/AN, 54.6; Hispanic, 50.3; White, 43.0; and Asian or Pacific Islander (API), 34.5 (Figure 1). Compared with age-adjusted rates among White adults, rates were higher among Black (RR = 1.8), AI/AN (RR = 1.3), and Hispanic adults (RR = 1.2) (Supplementary Table, https://stacks.cdc.gov/view/cdc/121713) with some variation by influenza season. During most influenza seasons, age-adjusted hospitalization rates were highest among Black adults, ranging from 1.5 to 2.4 times the rates among White adults. During the 2011–2012 and 2021–22 seasons, the highest influenza-associated hospitalization rates were among AI/AN adults, with age-adjusted rates 2.7 times those in White adults. Age-adjusted hospitalization rates among Hispanic adults were 2.1 times those among White adults in 2009–10 and 2021–22 (Figure 2) (Supplementary Table, https://stacks.cdc.gov/view/cdc/121713). In every season except 2011–12, API adults had the lowest hospitalization rates among all racial and ethnic groups, from 60% to 90% of those among White adults.

**FIGURE 3:**
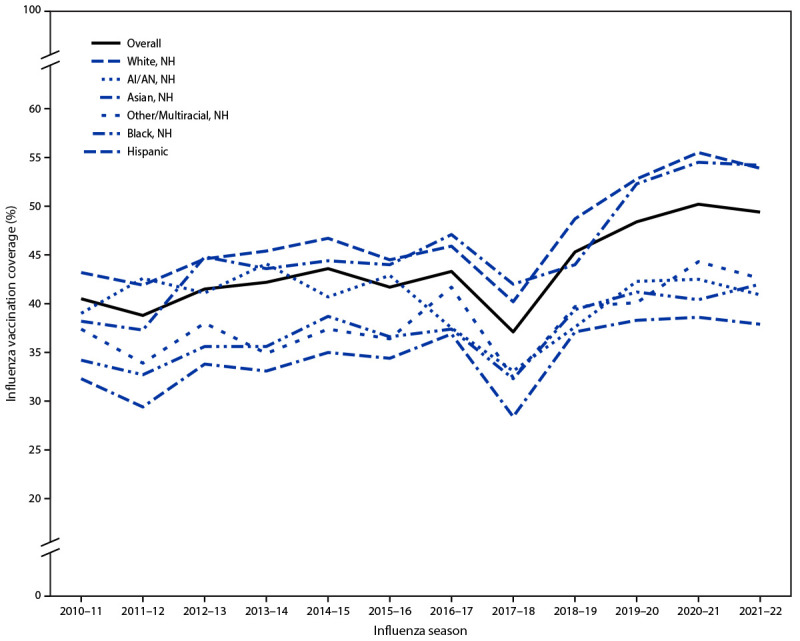
Influenza vaccination coverage among adults aged ≥18 years, by race and ethnicity and influenza season — Behavioral Risk Factor Surveillance System, United States, 2010–11 through 2021–22 **Abbreviations:** AI/AN = American Indian or Alaska Native; NH = non-Hispanic.

Overall vaccination coverage in the 2021–22 influenza season was 49.4% among adults aged ≥18 years and varied by race and ethnicity. Coverage was higher among White adults (53.9%) than among AI/AN (40.9%), Hispanic (37.9%), Black (42.0%), and multiracial and adults of other races (42.6%) and was similar to that among Asian adults (54.2%) (Table). From the 2010–11 through 2021–22 seasons, overall adult influenza vaccination coverage increased from 40.5% to 49.4% and increased within all racial and ethnic groups except AI/AN adults (Figure 3). Between the 2020–21 and 2021–22 seasons, coverage decreased among all adults by 0.8 percentage points and among White adults by 1.6 percentage points. In all other racial and ethnic groups, coverage was stable during the 2018–19 through 2021–22 influenza seasons. Since the 2010–11 influenza season, coverage has been consistently higher among White and Asian adults compared with that among Black and Hispanic adults.

**TABLE:** Influenza vaccination coverage, by race and ethnicity and demographic and access-to-care variables — Behavioral Risk Factor Surveillance System, United States, 2021–22 influenza season*

Characteristic	Race or ethnicity^†^														
	Overall		White		Black		Hispanic		Asian		AI/AN		Other/Multiple races		
	Sample no. (weighted %)	Influenza vaccination coverage % (95% CI)	Sample no. (weighted %)	Influenza vaccination coverage, % (95% CI)	Sample no. (weighted %)	Influenza vaccination coverage, % (95% CI)	Sample no. (weighted %)	Influenza vaccination coverage, % (95% CI)	Sample no. (weighted %)	Influenza vaccination coverage, % (95% CI)	Sample no. (weighted %)	Influenza vaccination coverage, % (95% CI)		Sample no. (weighted %)	Influenza vaccination coverage, % (95% CI)
**Overall**	319,846 (100.0)	49.4 (49.0–49.9)	239,619 (63.6)	53.9 (53.4–54.4)	25,046 (12.2)	42.0 (40.6–43.6)^§^	25,032 (15.6)	37.9 (36.3–39.5)^§^	8,035 (4.1)	54.2 (51.5–57.0)	5,250 (1.1)	40.9 (37.0–45.1)^§^	9,095 (3.4)		42.6 (39.8–45.5)^§^
**Age group, yrs**															
18−49	116,719 (52.9)	37.1 (36.5–37.8)^¶^	75,723 (45.1)	39.6 (38.9–40.4)^¶^	9,969 (55.5)^§^	29.4 (27.5–31.3)^§,¶^	16,024 (72.5)^§^	32.0 (30.2–33.9)^§,¶^	5,212 (78.9)^§^	52.1 (48.9–55.4)^§,¶^	2,279 (56.5)^§^	32.5 (27.5–38.1)^§,¶^	4,750 (68.4)^§^		37.1 (33.5–40.9)^¶^
50−64	85,144 (24.6)	52.4 (51.5–53.3)^¶^	64,980 (26.7)	54.2 (53.2–55.3)^¶^	7,319 (26.6)	50.5 (47.8–53.2)^§,¶^	5,559 (18.4)^§^	47.0 (43.5–50.7)^§,¶^	1,506 (14.2)^§^	55.5 (49.6–61.5)^§^	1,692 (27.7)	40.5 (34.2–47.4)^§,¶^	2,156 (18.1)^§^		45.5 (40.7–50.6)^§,¶^
≥65 (Ref)	117,983 (22.5)	73.9 (73.1–74.8)	98,916 (28.2)	75.7 (74.8–76.5)	7,758 (17.9)^§^	67.8 (64.4–71.1)^§^	3,449 (9.1)^§^	65.2 (60.5–69.9)^§^	1,317 (7.0)^§^	79.4 (74.4–84.0)	1,279 (15.8)^§^	76.4 (65.7–85.8)	2,189 (13.5)^§^		68.8 (62.6–74.8)^§^
High-risk condition** (among persons aged 18–64 yrs)															
Yes (Ref)	53,244 (24.4)	50.4 (49.3–51.6)	37,567 (25.1)	52.6 (51.2–53.9)	5,189 (28.2)^§^	47.0 (43.7–50.4)^§^	4,852 (21.5)^§^	46.6 (43.1–50.3)^§^	1,001 (12.7)^§^	53.6 (46.6–60.9)	1,398 (32.2)^§^	43.8 (36.1–52.3)^§^	2,039 (26.3)^§^		46.0 (40.4–52.0)^§^
No	145,674 (75.6)	39.4 (38.8–40.1)^¶^	101,444 (74.9)	42.6 (41.9–43.3)^¶^	11,829 (71.8)^§^	32.2 (30.4–34.1)^§,¶^	16,274 (78.5)^§^	32.3 (30.5–34.2)^§,¶^	5,545 (87.3)^§^	52.5 (49.4–55.7)^§^	2,497 (67.8)^§^	31.7 (27.2–36.7)^§,¶^	4,734 (73.7)		36.8 (33.1–40.7)^§,¶^
**Education level**															
High school or less	98,563 (39.8)	40.4 (39.5–41.2)^¶^	67,326 (35.0)	43.8 (42.8–44.9)^¶^	9,455 (45.3)^§^	38.5 (36.1–41.0)^§,¶^	12,796 (58.9)^§^	32.9 (30.9–35.0)^§,¶^	1,379 (21.4)^§^	50.1 (43.5–57.0)	2,273 (50.9)^§^	36.6 (31.2–42.7)^§,¶^	2,976 (38.8)^§^		32.5 (28.2–37.2)^§,¶^
Some college or technical school	88,077 (23.8)	47.5 (46.6–48.4)^¶^	66,866 (24.2)	50.2 (49.2–51.1)^¶^	7,082 (25.3)^§^	43.7 (40.6–46.8)^§^	5,850 (21.0)^§^	41.6 (38.0–45.3)^§,¶^	1,482 (16.8)^§^	49.5 (43.5–55.8)^¶^	1,724 (29.1)^§^	43.8 (37.2–51.0)	2,970 (28.1)^§^		42.0 (36.7–47.7)^§,¶^
College (Ref)	132,098 (36.5)	60.2 (59.5–60.9)	104,892 (40.8)	64.6 (63.8–65.4)	8,425 (29.4)^§^	46.0 (43.7–48.3)^§^	6,232 (20.0)^§^	47.2 (44.1–50.3)^§^	5,122 (61.8)^§^	57.1 (53.7–60.5)^§^	1,237 (20.0)^§^	49.2 (39.7–59.6)^§^	3,122 (33.1)^§^		55.2 (50.5–59.9)^§^
**U.S. Census Bureau Region^††^**															
Northeast (Ref)	68,359 (19.4)	55.5 (54.4–56.6)	52,959 (19.9)	60.2 (58.9–61.6)	3,791 (17.2)^§^	45.0 (41.4–48.7)^§^	5,869 (18.3)^§^	43.4 (40.4–46.5)^§^	1,935 (25.5)^§^	55.4 (51.2–59.8)^§^	412 (10.9)^§^	43.5 (29.8–60.1)^§^	1,538 (14.7)^§^		56.5 (48.8–64.4)
Midwest	84,306 (22.9)	51.3 (50.5–52.2)^¶^	69,655 (27.8)	54.1 (53.2–55.1)^¶^	4,427 (16.8)^§^	42.3 (39.0–45.7)^§^	3,828 (10.7)^§^	38.9 (35.4–42.5)^§^	1,222 (16.6)^§^	53.3 (48.0–58.7)	1,793 (20.9)^§^	45.9 (37.4–55.4)	1,704 (18.8)^§^		38.2 (32.6–44.5)^§,¶^
South	93,057 (38.8)	45.5 (44.7–46.3)^¶^	63,694 (35.5)	50.5 (49.6–51.4)^¶^	15,506 (58.8)^§^	41.5 (39.6–43.5)^§^	6,649 (40.7)^§^	33.8 (31.3–36.5)^§,¶^	1,510 (26.4)^§^	52.7 (46.3–59.4)	1,132 (40.7)^§^	35.2 (29.1–42.0)^§^	2,366 (36.2)		37.3 (32.6–42.5)^§,¶^
West	74,124 (19.0)	49.4 (48.3–50.5)^¶^	53,311 (16.9)	53.8 (52.6–55.0)^¶^	1,322 (7.1)^§^	37.3 (31.4–44.0)^§,¶^	8,686 (30.3)^§^	40.4 (37.4–43.5)^§^	3,368 (31.4)^§^	54.7 (49.7–60.0)	1,913 (27.5)^§^	45.4 (39.0–52.2)^§^	3,487 (30.3)^§^		46.9 (42.4–51.7)^§,¶^
**Medical insurance**															
Yes (Ref)	291,594 (91.5)	52.7 (52.2–53.2)	223,925 (95.2)	56.2 (55.7–56.8)	22,374 (91.9)^§^	44.3 (42.7–45.9)^§^	18,676 (75.4)^§^	43.5 (41.7–45.5)^§^	7,314 (95.4)	57.0 (54.1–59.9)	4,636 (90.8)^§^	44.8 (40.4–49.4)^§^	8137 (91.6)^§^		45.6 (42.6–48.7)^§^
No	16,195 (8.5)	17.6 (16.2–19.1)^¶^	7,985 (4.8)	13.9 (12.4–15.5)^¶^	1,401 (8.1)^§^	18.1 (14.5–22.5)^¶^	5,130 (24.6)^§^	21.2 (18.5–24.2)^§,¶^	285 (4.6)	22.0 (14.7–32.2)^¶^	381 (9.2)^§^	8.7 (5.2–14.4)^§,¶^	541 (8.4)^§^		17.8 (8.2–36.0)^¶^
**Personal health care provider**															
Yes (Ref)	278,538 (82.8)	54.8 (54.2–55.3)	214,307 (87.0)	58.3 (57.8–58.9)	21,966 (84.2)^§^	45.9 (44.2–47.5)^§^	17,730 (67.4)^§^	46.1 (44.1–48.1)^§^	6,499 (77.7)^§^	59.2 (56.1–62.4)	4,174 (77.5)^§^	45.1 (40.4–50.0)^§^	7,542 (78.9)^§^		48.3 (45.3–51.4)^§^
No	38,567 (17.2)	23.9 (22.8–24.9)^¶^	23,624 (13.0)	24.9 (23.7–26.1)^¶^	2,856 (15.8)^§^	21.9 (18.5–25.8)^¶^	7,004 (32.6)^§^	20.7 (18.4–23.1)^§,¶^	1,365 (22.3)^§^	36.9 (32.1–42.2)^§,¶^	1,001 (22.5)^§^	25.9 (19.9–33.3)^¶^	1,453 (21.1)^§^		23.4 (16.8–32.1)^¶^
**Yrs since last routine checkup**															
<1 (Ref)	251,361 (76.1)	56.6 (56.0–57.1)	190,656 (77.1)	61.2 (60.6–61.8)	21,124 (82.2)^§^	46.2 (44.6–48.0)^§^	17,388 (69.9)^§^	46.1 (44.1–48.1)^§^	5,775 (70.5)^§^	60.6 (57.3–64.0)	3,929 (73.2)^§^	48.7 (43.8–53.8)^§^	6,643 (71.4)^§^		49.7 (46.3–53.2)^§^
≥1	62,589 (23.9)	28.4 (27.6–29.3)^¶^	45,183 (22.9)	30.8 (29.9–31.8)^¶^	3,572 (17.8)^§^	23.0 (20.1–26.3)^§,¶^	6,810 (30.1)^§^	21.7 (19.6–24.1)^§,¶^	1,998 (29.5)^§^	39.9 (35.2–45.0)^§,¶^	1,177 (26.8)^§^	23.8 (17.8–31.3)^§,¶^	2,239 (28.6)^§^		27.6 (22.6–33.3)^¶^

**Abbreviations:** AI/AN = American Indian or Alaska Native; Ref = referent group.

* Data from respondents interviewed during September 2021–June 2022 were analyzed to estimate influenza vaccination received during July 2021–May 2022. Influenza vaccination coverage estimates are based on a sample size of 291,839; 28,007 respondents were excluded from analyses of vaccination coverage because of missing influenza vaccination status.

^†^ White, Black, Asian, AI/AN, and other/multiple race persons are non-Hispanic; Hispanic persons could be of any race.

^§^ p<0.05 when compared with non-Hispanic White (column comparisons).

^¶^ p<0.05 when compared with referent category (row comparisons).

** Includes people with asthma, diabetes, heart disease, chronic obstructive pulmonary disease, or cancers other than skin cancer.

^††^ https://www2.census.gov/geo/pdfs/maps-data/maps/reference/us_regdiv.pdf

During the 2021–22 influenza season, vaccination coverage among all racial and ethnic groups was higher among adults aged ≥65 years than among younger adults and among the following groups: those with medical insurance compared with those without medical insurance; those who had a personal health care provider compared with those without a personal health care provider; and those who had had a routine medical checkup in the past year compared with those who had not. However, compared with White adults, Hispanic adults were less likely to have medical insurance, and Hispanic, AI/AN, and multiracial and adults of other races were less likely to have a personal health care provider and a medical checkup in the past year. In addition, among adults with medical insurance, a personal health care provider, and a routine medical checkup in the past year, and in most age and education strata, influenza vaccination coverage was higher among White adults than among Black, Hispanic, AI/AN, and multiracial and adults of other races (Table).

## Discussion

Racial and ethnic disparities in influenza-associated hospitalizations were consistently observed among Black, AI/AN, and Hispanic adults compared with White adults, with hospitalization rates an average of 1.2 to 1.8 times those in White adults during the past 13 seasons. Similar disparities have been observed for COVID-19 hospitalizations (*8*). The reasons for these disparities in severe respiratory disease are likely multifactorial. Influenza vaccination coverage continues to be lower among Black, AI/AN, and Hispanic adults compared with coverage among White and Asian adults. Distrust of the medical system, misperceptions about vaccine safety, and higher levels of concern about side effects have contributed to lower coverage (*9*). Members of racial and ethnic minority groups might face barriers to affordable, quality health care, including access to health insurance, transportation to health providers, and child care; therefore, they might have fewer opportunities for preventive health care and increased vulnerability to chronic medical conditions (*10*). Higher prevalences of chronic medical conditions have been independently associated with more severe influenza outcomes (*11*,*12*), and downstream effects of structural racism have been demonstrated to affect economic stability, housing, and education (*10*,*13*,*14*). In addition, poverty, crowded housing, and community exposure to respiratory diseases are associated with more severe influenza disease (*15*,*16*).

In contrast to the decline in influenza vaccination observed among children during the COVID-19 pandemic (*6*), recent coverage among adults has not decreased compared with prepandemic estimates. However, longstanding disparities in coverage by race and ethnicity remain. The finding that adults of some minority racial and ethnic groups were less likely than White adults to have medical insurance and a personal health care provider suggests that access to influenza vaccination likely plays a role in lower coverage among these groups. Racial and ethnic disparities in COVID-19 vaccination coverage that were evident early in the COVID-19 vaccination program have decreased or been eliminated over time, likely related to efforts by immunization programs to provide equitable access to COVID-19 vaccination, such as making vaccines available free of charge at varied and nontraditional locations (*17*,*18*). However, disparities in COVID-19 booster vaccination are now evident, and differences in influenza vaccination coverage within most socioeconomic and access-to-care strata suggest that in addition to access limitations, other factors contributed to disparities in coverage. A provider recommendation and offer of vaccination is strongly associated with vaccination (*19*). BRFSS does not collect information on receipt of provider recommendations or offers of vaccination; however, variables such as having a medical checkup in the past year and having a personal health care provider can serve as proxies for these data. Overall, adults who reported having a medical checkup in the past year were twice as likely to be vaccinated as those who did not. Hispanic, AI/AN, and multiracial and adults of other races were less likely than were White adults to report having a personal health care provider and a routine medical checkup in the past 12 months. Moreover, even among Black, Hispanic, AI/AN, and multiracial and adults of other races who reported a recent medical checkup, influenza vaccination coverage was <50% and was also lower than coverage among White adults with a recent medical checkup, suggesting that missed opportunities for influenza vaccination occurred during these visits. Following the standards for adult immunization practice, providers should assess patient vaccination status at all medical visits and offer (or provide a referral for) all recommended vaccines (*20*). Meeting this standard in a culturally responsive manner could help reduce observed disparities in vaccination coverage.

Programmatic efforts and communication campaigns, such as Partnering for Vaccine Equity: Equity in Adult vaccination, that have brought COVID-19 vaccines to communities through nontraditional settings (local libraries, local businesses [e.g., barber shops/salons, thrift stores, restaurants, and grocery stores], and school-based events) likely contributed to decreased disparities in COVID-19 vaccination and might also decrease disparities in influenza vaccination.^§§^ Surveys collected after 2 years of a tailored vaccination campaign^¶¶^ collaboratively led by the Ad Council, the American Medical Association, and CDC indicated that concerns about influenza vaccine risks or side effects were reduced from 43% to 33% among Black adults and from 41% to 32% among Hispanic adults.

The findings in this report are subject to at least seven limitations. First, because FluSurv-NET surveillance is conducted in selected counties within the United States, findings might not represent the entire U.S. population. Second, influenza-associated hospitalizations reported to FluSurv-NET are identified by clinician-directed testing; hospitalization rates might be underestimated, as they have not been adjusted for testing practices, which differ by surveillance site, age group, and timing during influenza seasons (*2*) and might also vary by race and ethnicity. Third, within FluSurv-NET data, approximately 17% of persons were missing ethnicity and were classified based only on their reported race; 7% were missing race. Fourth, weighting adjustments for BRFSS survey data used to assess influenza vaccination coverage might not eliminate all possible bias from incomplete sample frame because households with no telephones are excluded. Fifth, survey response rates were low, and influenza vaccination coverage might differ between survey respondents and nonrespondents; survey weighting adjustments might not adequately control for these differences. Sixth, influenza vaccination status was self-reported and subject to recall error and social desirability bias. Finally, errors in BRFSS data from incomplete sample frame, nonresponse, and accuracy of reported influenza vaccination status might change over time, which could lead to inaccurate assessment of trends in vaccination coverage.

The findings in this report highlight persistent disparities in influenza disease severity among adults in some racial and ethnic minority groups during 2009–2022, as well as continued disparities in influenza vaccination coverage among adults during the same period. Increasing influenza vaccination coverage among racial and ethnic minorities could reduce disparities in the risk for severe disease. National, state, and community-level efforts to build trust, increase access to vaccination services, and combat misinformation among racial and ethnic minority communities are important actions for increasing vaccination coverage in these groups. Interventions that support and promote partnerships at the community level to effectively reduce racial and ethnic disparities in influenza vaccination include creating and training (or partnering with) a network of local community trusted messengers reflecting the communities served; using trusted messengers to address misinformation and promote accurate, culturally responsive vaccine messages, including through social media; and working with culturally competent health care providers to provide a strong recommendation for influenza vaccination. National, tailored influenza vaccination campaigns can reinforce local efforts to increase awareness of the importance of influenza vaccination among target audiences to encourage increased vaccination coverage among these groups.

**Summary**

**What is already known about this topic?**

Historically, persons from some racial and ethnic minority groups have had higher rates of influenza hospitalization and death and lower influenza vaccination coverage than White persons.

**What is added by this report?**

Racial and ethnic disparities in influenza disease severity and vaccination coverage, along with disparities in access to care, have persisted since the 2009–10 and 2010–11 influenza seasons.

**What are the implications for public health practice?**

Tailored efforts to increase access to influenza vaccination and improve vaccine confidence among racial and ethnic minority communities, including creating culturally relevant communication campaigns and offering vaccination in nontraditional settings, are critical and might decrease disparities in influenza vaccination and disease severity.

## Footnotes

* Emerging Infections Program sites include selected counties in California, Colorado, Connecticut, Georgia, Maryland, Minnesota, New Mexico, New York, Oregon, and Tennessee, and FluSurv-NET sites include selected counties in Idaho (2009–10 to 2010–11 only), Iowa (2009–10, 2012–13, 2020–21, and 2021–22 only), Michigan and North Dakota (2009–10 only), Ohio (2010–11 to 2021–22), Oklahoma (2009–10 to 2010–11 only), Rhode Island (2010–11 to 2012–13 only), South Dakota (2009–10 only) and Utah (2010–11 to 2021–22). Across these seasons, approximately 25–29 million persons (8%–9% of the U.S. population) were in the FluSurv-NET catchment area.

† Period for the 2009–10 season was April 15, 2009, through April 30, 2010, and the period for 2021–22 was October 1, 2021, through June 11, 2022.

§
https://www.cdc.gov/nchs/nvss/bridged_race.htm

¶ For FluSurv-NET data, race and ethnicity were categorized using the National Center for Health Statistics categories as non-Hispanic White, non-Hispanic Black, Hispanic or Latino, non-Hispanic Asian or Pacific Islander, and non-Hispanic American Indian or Alaska Native. Persons of more than one race or of unknown race were excluded from the FluSurv-NET analysis because population denominators were not available for these groups. Persons of any race (including unknown race) but of Hispanic ethnicity were classified as Hispanic. In 17% of included persons (across all seasons), ethnicity was unknown, but race was known; these persons were assumed to be of non-Hispanic ethnicity. In the 2017–18 season, race and ethnicity were collected on a stratified random sample of hospitalized adult influenza patients aged ≥50 years, so sampling weights were used to calculate the weighted number of hospitalizations by race and ethnicity that season. For BRFSS data, race and ethnicity were categorized similarly in the vaccination coverage analyses with two exceptions: 1) non-Hispanic persons reporting more than one race were categorized along with persons reporting Native Hawaiian/Other Pacific Islander and “other” race into a “Non-Hispanic other/multiple race” category; and 2) persons of unknown ethnicity or race were excluded from the analysis (2.8% of BRFSS respondents in the 2021–22 season).

**
https://www.cdc.gov/brfss

†† 45 CFR. part 46.102[l][2], 21 CFR part 56; 42 USC Sect. 241[d]; 5 USC Sect. 552a; 44 USC Sect. 3501 et seq.

§§
https://www.cdc.gov/vaccines/health-equity/

¶¶
https://getmyflushot.org/
